# A General Consideration of Diseases of Ear, Nose and Throat

**Published:** 1899-11

**Authors:** Wm. J. Cox

**Affiliations:** Gainesville, Ga.


### A GENERAL CONSIDERATION OF DISEASES OF EAR, NOSE AND THROAT.

BY WM. J. COX, M.D., PH.G.,

GAINESVILLE, GA.

This is a very broad and comprehensive subject upon which to wield a short paper; but as diseases of these organs are so often associated in the same individual it is, perhaps, not amiss to consider them together.

It is not often that we find middle ear troubles without a general catarrhal condition of the nasopharyngeal mucous membrane. Perhaps in no class of diseases have there been more rapid strides in the past few years than in diseases of the middle-ear. And there is probably no class of diseases in which the physician can render more valuable service or at least arrest impending dangers than in nose and throat work.

There are several important etiological factors in the production of nasopharyngeal diseases. Among the most important are hereditary predisposition, improper nourishment in childhood, a lack of proper bathing and physical exercise, and close confinement to the house in school children, and, perhaps, the most important factor during the last few years have been the epidemics of la grippe. I find also that a great many people who suffer with this class of disease are of a nervous temperament and of a uric acid diathesis. You will frequently find them suffering with nervousness, melancholia, and indigestion. In fact, a great many of them are the subjects of gastric catarrh with hyperacidity of the gastric juice, and a general relaxed condition of the muscular system. Hence the importance of a well-selected general treatment in connection with local measures.

These troubles usually begin in children with adenoids of the post-nasal space and hypertrophy of the pharyngeal and faucial tonsil, and later hypertrophy of the turbinates and extension of the catarrhal condition through the Eustachian tube to the middle ear.

In my office I have a compressed spray apparatus and galvano-cautery, and find with the proper use of sprays and cauteries, together with the proper general treatment, most patients make a very rapid improvement. Especially is this true of patients coming from a lower altitude.

I generally use the compressed spray for opening up the Eustachian tube, which I usually find not very difficult after the hypertrophies have been removed. Of course in old and atrophic conditions the prognosis is not so good, and it is with a great deal more difficllty that we are enabled to relieve this class of patients.

The general practitioner should especially be interested in recognizing these troubles early, and, also, in the method of prevention. I do not know of any field where one can render more valuable service to humanity than recognizing these troubles early and thereby prevent a general catarrhal condition of stomach, bronchi, and, perhaps, tuberculosis. This is the age of progressive medicine, and he who prevents disease deserves a higher place in the realms of the profession than he who cures it.

In conclusion let me say that when you begin to notice children complaining of so-called "growing pains," who are nervous and irritable, who do not sleep well at night, who breathe loudly, if they have any one or all of these symptoms, examine and you will find some throat and nasal trouble.

*Arlington Hotel.*

